# Ligand Induced Conformational Changes of the Human Serotonin Transporter Revealed by Molecular Dynamics Simulations

**DOI:** 10.1371/journal.pone.0063635

**Published:** 2013-06-12

**Authors:** Heidi Koldsø, Henriette Elisabeth Autzen, Julie Grouleff, Birgit Schiøtt

**Affiliations:** 1 The Center for Insoluble Protein Structures (*in*SPIN) and the Interdisciplinary Nanoscience Center (*i*NANO), Aarhus University, Aarhus, Denmark; 2 Department of Chemistry, Aarhus University, Aarhus, Denmark; University of Bologna & Italian Institute of Technology, Italy

## Abstract

The competitive inhibitor cocaine and the non-competitive inhibitor ibogaine induce different conformational states of the human serotonin transporter. It has been shown from accessibility experiments that cocaine mainly induces an outward-facing conformation, while the non-competitive inhibitor ibogaine, and its active metabolite noribogaine, have been proposed to induce an inward-facing conformation of the human serotonin transporter similar to what has been observed for the endogenous substrate, serotonin. The ligand induced conformational changes within the human serotonin transporter caused by these three different types of ligands, substrate, non-competitive and competitive inhibitors, are studied from multiple atomistic molecular dynamics simulations initiated from a homology model of the human serotonin transporter. The results reveal that diverse conformations of the human serotonin transporter are captured from the molecular dynamics simulations depending on the type of the ligand bound. The inward-facing conformation of the human serotonin transporter is reached with noribogaine bound, and this state resembles a previously identified inward-facing conformation of the human serotonin transporter obtained from molecular dynamics simulation with bound substrate, but also a recently published inward-facing conformation of a bacterial homolog, the leucine transporter from *Aquifex Aoelicus*. The differences observed in ligand induced behavior are found to originate from different interaction patterns between the ligands and the protein. Such atomic-level understanding of how an inhibitor can dictate the conformational response of a transporter by ligand binding may be of great importance for future drug design.

## Introduction

**Figure 1:**
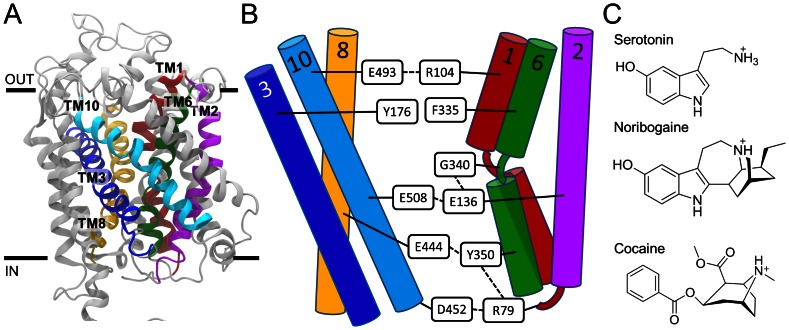
Homology model of hSERT, outline of important interactions and chemical structures of ligands. **A**) Homology model of hSERT [8], [9]. The transporter is shown in grey with TM1 (red), TM2 (magenta), TM3 (dark blue), TM6 (green), TM8 (yellow) and TM10 (cyan) highlighted. Serotonin is shown in purple sticks in the central binding pocket. **B**) Overview of important interactions between the scaffold and bundle (represented by TM3, 8, 10 and TM1, 2, 6 respectively). The dashed lines indicate hydrogen bonds, salt bridge interaction or cation-π interactions. **C**) Chemical structures of three hSERT ligands: serotonin (substrate), noribogaine (non-competitive inhibitor) and cocaine (competitive inhibitor).

The human serotonin transporter (hSERT) belongs to the family of monoamine transporters, and it consists of 12 transmembrane α-helices (TM). The transport of the substrate, serotonin (5-HT, 5-hydroxytryptamine) in hSERT proceeds by an alternating access mechanism [1]–[4]. The molecular events involved in the alternating access mechanism of hSERT have been suggested from a bacterial homologue, the leucine transporter (LeuT) from *Aquifex aoelicus* [5]. The central binding pocket of LeuT is composed of TM1, TM3, TM6, and TM8 [5]. Based on molecular modeling and accessibility measurements it was proposed that the relative movement of a four-helix bundle consisting of TM1, TM2, TM6, and TM7 with respect to a scaffold (consisting of TM3–5 and TM8–10) constitutes the transport mechanism of the neurotransmitter sodium symporter (NSS) family of transporters [3], as seen in Figure 1a. In this mechanism, rocking of the bundle towards the scaffold results in the transporter being open either to the extracellular or the intracellular space [3]. TM1 and TM6 are partly unwound at the central part of the helix, which allows for these two helices to move in a hinge-type of motion. Molecular interactions between residues in the bundle and scaffold, respectively, are schematically depicted in Figure 1b, and includes the aromatic lid, Tyr176 and Phe335 in hSERT, screening the ligand binding site from the extracellular side, as well as two hydrogen bonding networks extending at the extracellular and intracellular side, respectively, from the central binding site. However, atomic resolution details of the molecular and structural events associated with substrate translocation in the human monoamine transporters still remains to be fully understood due to lack of high resolution structures of mammalian transporters. Despite a rather low sequence identity of 20–25%, LeuT has proven to be a reliable template for modeling of the human monoamine transporters [6]–[18]. Since the first crystal structure of LeuT emerged in 2005 [5] several others have been solved but until recently the states captured in the crystal structures were minor variations of the outward-facing conformation [19]–[25]. A crystal structure of an engineered LeuT was published in 2012 revealing LeuT in an inward-facing conformation [25]. This previously unresolved conformation of LeuT revealed one dominating movement during transport, namely the movement of the intracellular half of TM1, TM1a, from the four-helix bundle [25]. A concerted motion of the bundle was indeed observed with respect to the scaffold; however an additional internal hinge-type motion of TM1a was detected with respect the remaining part of the bundle, allowing for a much more dynamic TM1a than the rest of the bundle [25]. This behavior is similar to what we have previously identified from molecular dynamics (MD) simulations of hSERT where a transformation of an originally outward-facing occluded state of the protein with bound serotonin to an inward-facing state was found to be associated with a larger movement of TM1a than TM1b [7].

hSERT is the target of many antipsychotic pharmaceuticals but also of illicit drugs. Addiction to cocaine and other psychostimulants represents a worldwide problem with both great human and societal costs [26], [27]. Cocaine is a potent, non-selective, competitive inhibitor of hSERT [28]. As other competitive inhibitors of hSERT, cocaine hinders reuptake of serotonin by blocking the primary binding site and inducing an extracellular-facing state of the transporter, a mechanism that has also been proposed for the interaction between cocaine and other psychostimulant and the human dopamine transporter (hDAT) [13], [29]. Ibogaine, a natural occurring plant alkaloid from the West African shrub *Tabernanthe iboga*, has been alleged to be effective in treatment of drug abuse [30]–[33] and it has been established that ibogaine inhibits hSERT non-competitively [34]. It has elegantly been demonstrated that the accessibility of reactive substituted cysteines in the intracellular pathway increase upon ibogaine binding and accordingly it has been proposed that ibogaine stabilizes hSERT in an inward-facing conformation hereby rendering the binding site less accessible to substrate binding from the extracellular side. Initially the binding site of ibogaine was proposed to overlap with that of the natural substrate, serotonin [34], partly due to its structural similarity to serotonin and partly due to its ability to induce the same conformation of hSERT as 5-HT. However, a recent study indicates that the binding sites of 5-HT and ibogaine may not necessarily overlap, and it was suggested that ibogaine binds at an extracellular surface exposed site that forms upon formation of the inward-facing conformation [35]. *In vivo*, ibogaine undergoes demethylation into 12-hydroxyibogamine (noribogaine), which is structurally even more similar to serotonin than ibogaine. Noribogaine has about ten times greater affinity towards hSERT and a longer metabolic half-life than ibogaine, which suggests that noribogaine is the active compound of the two [36]. Moreover, because the structure of noribogaine essentially contains the structure of serotonin, it is an obvious assumption that their binding modes in hSERT are similar. [37] Yet, serotonin is transported while noribogaine hinders transport [36] but induces an opening of an inward-facing channel as evidenced from accessibility data [3], [34].

In this study, we explore by MD simulations the differences in dynamic behavior of hSERT in response to the binding of a substrate (serotonin), a competitive (cocaine) and a non-competitive inhibitor (noribogine), all being initially located in the central substrate binding site. Chemical structures of serotonin, noribogaine and cocaine are provided in Figure 1c. From extended MD simulations it is observed that the non-competitive inhibitor noribogaine is able to induce an inward-facing state of the transporter as also determined from experiments. A comparable behavior of hSERT is induced by the substrate similar to previous observations [7], while the competitive inhibitor cocaine favors the outward-facing conformation of hSERT. We describe how the different ligands affect the interactions between the scaffold and bundle of hSERT. These observations allow us to gain a deeper understanding of the transport mechanism of hSERT and to reveal important differences in the mechanism of action between a competitive and a non-competitive inhibitor. Such knowledge may be valuable for the future development of high affinity anti-abuse drugs.

## Materials and Methods

### Homology modeling

The homology model of hSERT was constructed using MODLLER 9v5 [38], [39] utilizing the outward-occluded LeuT structure (PDB code 2A65 [5]) as a template and including modeling of the long extracellular loop 2 (EL2) as described previously [8], [9]. Briefly, the homology model was selected based on different criteria; a large substrate binding cavity, good coordination of the sodium ions, and a conformation of EL2 complying with experimental knowledge of *e.g*. glucosylation sites, proteolysis sites and the formation of a disulfide bridge [9].

### Ligand preparation

The docking of serotonin to the central binding pocket has previously been performed and experimentally validated [8], [12]. Cocaine was prepared for modeling from a crystal structure [40], and noribogaine was built in Maestro 9.1 [41]. The amine groups of noribogaine and cocaine were modeled as charged in accordance with pK_a_ predictions with Epik 2.1 [42], [43] at physiological pH. For cocaine, in which this quaternary ammonium constitutes a chiral center, only the R-enantiomer resulted in docking poses containing a direct salt bridge between the nitrogen atom and Asp98 and with an orientation similar to the previously validated cocaine binding mode in hDAT [13]. The ligands were minimized until convergence and the minimized structures served as input for a Low-Mode Conformational Search [44] in order to obtain the global minimum conformations. Both the minimization and the conformational search were performed in MacroModel 9.8 [45] using the OPLS_2005 [46] FF and implicit water. The lowest energy structures from the conformational searches were used as input for the docking calculations.

### Induced fit docking (IFD)

The ligands were docked according to the IFD protocol developed by Schrödinger (Schrödinger LLC) [47], [48]. The energy window was increased to 50 kcal/mol in the filtering stage in each case to allow for more poses. For IFD of cocaine, the default hydrogen bond cut-off was increased from −0.05 kcal/mol to 0.00 kcal/mol, thereby removing the criterion of a minimum of one hydrogen bond in a reported pose. Without removal of this constraint, it was not possible for cocaine to bind fully inside the primary pocket of hSERT, which was expected based on observations made for cocaine binding to hDAT [13]. Furthermore, in the docking of noribogaine the side-chain conformations of Asp98 were not sampled in order to conserve the coordination between this residue and Na1, which has been shown to be present in hSERT [7], [9], [12], [18]. For the noribogaine and cocaine docking calculations, the binding site was defined from the serotonin molecule already bound inside the homology model of hSERT [8]. The SP scoring function [49] was applied in the initial docking stage whereas the XP scoring function [50] was used in the final docking in all IFD calculations. The poses were visually clustered based on a maximal root-mean-square-deviation (RMSD) of 2.0 Å of selected non-hydrogen atoms of the ligands within each cluster. Furthermore, poses in which the ligand was not fully situated inside the central cavity binding site were not considered further in this study. A table of all noribogaine and cocaine poses can be found in the Supporting Information (Table S1 +S2).

### Vestibular pocket IFD

IFD dockings of serotonin into the vestibular binding site, the S2 site, was done using selected conformations of hSERT extracted from the MD simulations, and were performed following the same protocol as described above. Prior to the docking calculations the snapshot structure of hSERT from the simulation trajectory was subjected to 10,000 steps of conjugate gradient minimization and prepared with the Protein Preparation Wizard in the Schrödinger 2010 suite [51]. Further details can be found in Supporting Information S1.

### Molecular dynamics simulations

All MD simulations were carried out using NAMD [52] using the OPLS force field [53], [54] due to the availability of parameters for cocaine and noribogaine in this force field [46]. OPLS_2005 parameters were extracted from IMPACT in the Schrödinger software package [46], [55]. As a control, the serotonin bound hSERT was also subject to simulations for comparison to the earlier reported trends observed using the CHARMM force field [56]. Each protein-ligand complex was modeled as a dimer. The dimer was built according to the LeuT dimer as reported in the crystal structure [5]. Similar dimer structures have previously been applied in simulations of LeuT [57], [58] and hSERT [7], [10]. Since hSERT has experimentally been observed to function only when found at least as a dimer in cell membranes [59], [60] this seems to be a reasonable choice. Simulating a dimeric system furthermore allows for better statistics by doubling the number of protein-ligand complexes sampled. The hSERT dimers were inserted into a pre-equilibrated POPC membrane patch using an internally adapted version of the membrane builder in VMD [61] and applying OPLS_2005 parameters extracted in same way as for the ligands [46]. The systems were solvated with water molecules using the TIP3P water model [62], and finally, neutralized by addition of NaCl to an ionic concentration of 0.2 M in order to mimic a physiological salt concentration. The non-bonded parameters of the chloride and sodium ions were similarly extracted from IMPACT in the Schrödinger suite [46], [55]. The three simulation systems had dimension of approximately 95 Å×130 Å×120 Å and contained ∼120,000 atoms, including ∼190 POPC molecules, ∼26000 TIP3P water molecules, ∼290 sodium ions and ∼310 chloride ions (the exact numbers are given in Supporting Information S1).

Prior to the simulations, the systems were subjected to a conjugate gradient minimization step to prevent atom-atom overlap at the dimer interface. The minimizations were run using NAMD version 2.6 [52]. Following minimization, each system was equilibrated in NAMD 2.7 [52] for 2.5 ns in two stages; during the first 0.5 ns, everything but the lipid tails was held fixed (*NVT* ensemble, T = 310 K), allowing the lipid tails to adapt to the protein; in the next 2 ns, the full system was subjected to a restraint free simulation (*NPT* ensemble, T = 310K, P = 1 atm) allowing all parts of the system to mutually adapt to one another. Three repeats of each of the three dimeric systems were run for 60 ns including the 2 ns restraint-free equilibration stage.

The temperature was controlled with Langevin dynamics, while the pressure was controlled by the Nosé-Hoover Langevin piston method [63], [64]. The PME method was used for full electrostatics [65], and short-range interactions were truncated at a cut-off of 12 Å, using a switching function from 10 Å. The pair-list, containing all the pairs of atoms for which non-bonded interactions are calculated, included atoms within 14 Å and was updated after every 20 time steps. The bonded interactions were calculated every 1 fs time step, and a time step of 2 and 4 fs was used for calculating the short-range non-bonded interactions and long-range electrostatics with PME, respectively. Snapshots were saved every 1 ps of which 1,500 were used for analysis.

## Results and Discussion

In this study, equilibrium all-atom simulations were performed of a hSERT homology model containing three distinctly different types of ligands within the central binding pocket. It has been shown from substituted cysteine accessibility experiments that the competitive inhibitor cocaine induces an outward-facing conformation of hSERT [3], [34], while the non-competitive inhibitor ibogaine has been seen to induce an inward-facing conformation based on solvent accessibility data of residues placed in the putative intracellular pathway [3], [34]. The substrate serotonin must inevitably also induce an inward-facing conformation, before being released to the cytoplasmic space followed by protein reorientation back to an outward-facing conformation. We have previously studied substrate induced conformational changes of hSERT [7], and in the present study we explore the differences in transporter conformations depending on the type of ligand bound in the central binding pocket.

**Figure 2:**
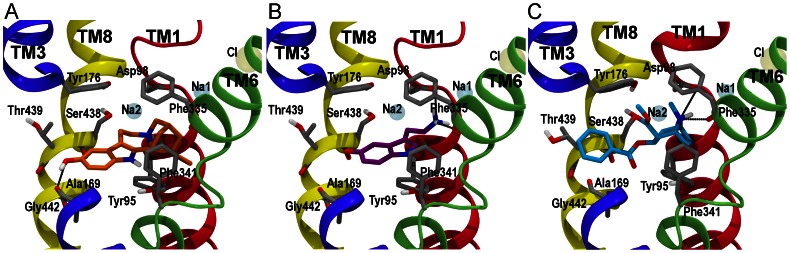
Binding of Noribogaine, serotonin and cocaine to the central binding pocket of hSERT. The transmembrane helices that constitute the central binding site, TM1 (red), TM3 (blue), TM6 (green) and TM8 (yellow) are shown in cartoon and the side chains of central amino acid residues belonging to these helices are shown in grey sticks. Residues 171 to 174 in TM3 have been omitted for clarity. The ions are displayed as transparent spheres, the sodium ions in cyan and the chloride ion in yellow. **A**) The selected binding mode of noribogaine within the primary binding site in hSERT. The ligand is shown in orange sticks. **B**) Biochemically validated binding mode of serotonin (purple) in hSERT [12]. **C**) Binding mode of cocaine (cyan) observed within the primary binding site of hSERT similar to the binding mode of cocaine in hDAT [13].

The validated binding mode of serotonin within hSERT [8] and the selected poses from the IFD calculations of noribogaine and cocaine to hSERT were used as the starting structures for the MD simulations. The initial binding modes of the ligands are displayed in Figure 2, from which it is obvious that the IFD procedure did not affect the protein conformation much (Figure S1). The simulations of hSERT with cocaine bound were initiated from a ligand binding mode similar to the one previously described by Beuming *et al.* [13]for cocaine binding to hDAT. Based on the great similarities within the central binding pocket of hDAT and hSERT [8], [66] and on a similar effect on ligand binding affinity of corresponding residues in hSERT and hDAT observed by mutagenesis [13], [29] we hypothesize that cocaine binds similarly to hSERT. Additionally, this binding mode was the only one observed within the central binding pocket of hSERT from the docking calculations and all poses within this binding mode were very closely related, as judged by the calculated RMSD-values, but also with respect to the GlideScores and accordingly the binding mode most closely resembling the validated binding mode in hDAT by Beuming *et al.* [13] was used (See Table S2). The simulations containing serotonin were initiated from the pose with the most favorable GlideScore and Emodel and belonging to the binding mode previously biochemically validated [12] possessing a salt-bridge interaction between the ammonium ion and Asp98 and with the 5-hydroxyl group harbored in a pocket formed by Ala169, Ser438 and Thr439 [8]. Since the structure of serotonin is inherent within the chemical structure of noribogaine, it is believed that the two ligands bind in a similar manner to hSERT [34], though another non-identified binding site has recently been proposed [35]. The docking pose of noribogaine from the IFD that resembles serotonin binding in hSERT [12] the most was also found to be the dominating binding mode and to possess the best overall score when looking at both GlideScore and Emodel and was therefore utilized as the starting point for the MD simulations, see Figure 2 and Table S1. Furthermore, this binding mode has recently been found to account for observations when mutating amino acids in the substrate binding site [37]. The simulations were initiated from a hSERT dimer with bound ligand in each monomer and embedded in a POPC lipid bilayer. A total simulation time of 60 ns was achieved, and three repeats for each ligand bound to dimeric hSERT were performed to allow for better sampling, since it has been observed to be an advantage with several simulations compared to one very long trajectory [7], [67]. Thus, three repeats of each system for 60 ns resulted in a total of six trajectories for analysis given the dimeric nature of hSERT and a total simulation time of 540 ns for the dimer hSERT system. The simulations of noribogaine, serotonin and cocaine are termed; ***NXa***, ***NXb***, ***SXa***, ***SXb***, ***CXa***, ***CXb*** (***X*** represents repeat 1, 2, and 3 whereas a/b refers to the two monomers making up the dimer, respectively).

The stability of the molecular systems was explored through root-mean-square deviation (RMSD) and fluctuation (RMSF) measurements. The RMSD of the C_α_ atoms of the monomers reached a value of 3 Å after a few nanoseconds of simulation (Figure S2 A–C), whereas the RMSD of the C_α_ atoms within the TM parts equilibrates to a value below 3 Å after a few nanoseconds of simulation (Figure S2 D–F), indicating stable membrane protein systems. The RMSF of the C_α_ atoms also indicates that the systems remain stable with the largest movements occurring in the loop regions (Figure S2 panel G–I). The RMSD of non-hydrogen atoms of the ligands with respect to the minimized structure furthermore indicates that the ligand in general remains stable in the binding pocket (Figure S2 panel J–L), though some movements are observed in a few of the trajectories, as discussed below.

**Figure 3:**
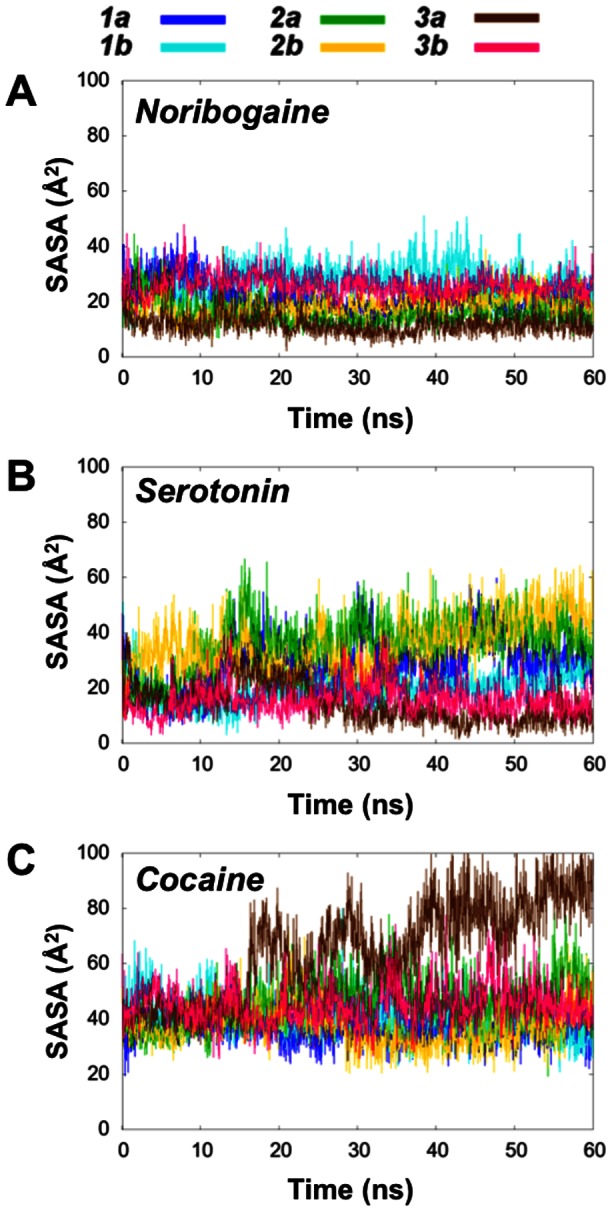
Solvent exposure of the aromatic lid in hSERT with noribogaine A), serotonin B) and cocaine C). **A**)–**C**). Plots of the SASA of the side chains of the aromatic lid, Phe335 and Tyr176, as it evolves during the six trajectories for each ligand.

Substituted cysteine accessibility experiments of hSERT have indicated that cocaine induces an outward-facing conformation of the transporter, while the substrate serotonin and the non-competitive inhibitor ibogaine, and most likely also its physiologically active metabolite noribogaine [68], all induce an inward-facing conformation [3], [34]. It is therefore of great interest to understand the ligand induced differences in conformational response of hSERT when different ligands are bound. It is known from experiments that the presence of a substrate in LeuT restrains the dynamics of the extracellular vestibule [69]. This is the same behavior that we observe regarding the stability of the interaction between the extracellular salt bridge pair Arg104 and Glu493 in hSERT (Figure S3). This direct ionic interaction is stable in all of the systems similar to what was observed upon substrate binding [7]. Since the study by Koldsø *et al.* [7] was performed with another force field than the one applied in this study, this ligand induced effect does not seem to be force field dependent [7]. To investigate the amount of extracellular gate opening, the time dependent solvent accessible surface area (SASA) of the aromatic lid located just above the central binding pocket was measured from all trajectories using VMD [61]. As appreciated from Figure 3, it is evident that the aromatic lid is most solvent exposed in the systems that contain cocaine, while the serotonin systems are intermediate and the noribogaine systems demonstrate the lowest degree of solvent accessibility from the extracellular side. Especially the cocaine system ***C3a*** stands out as the single most outwards-facing system.

**Figure 4:**
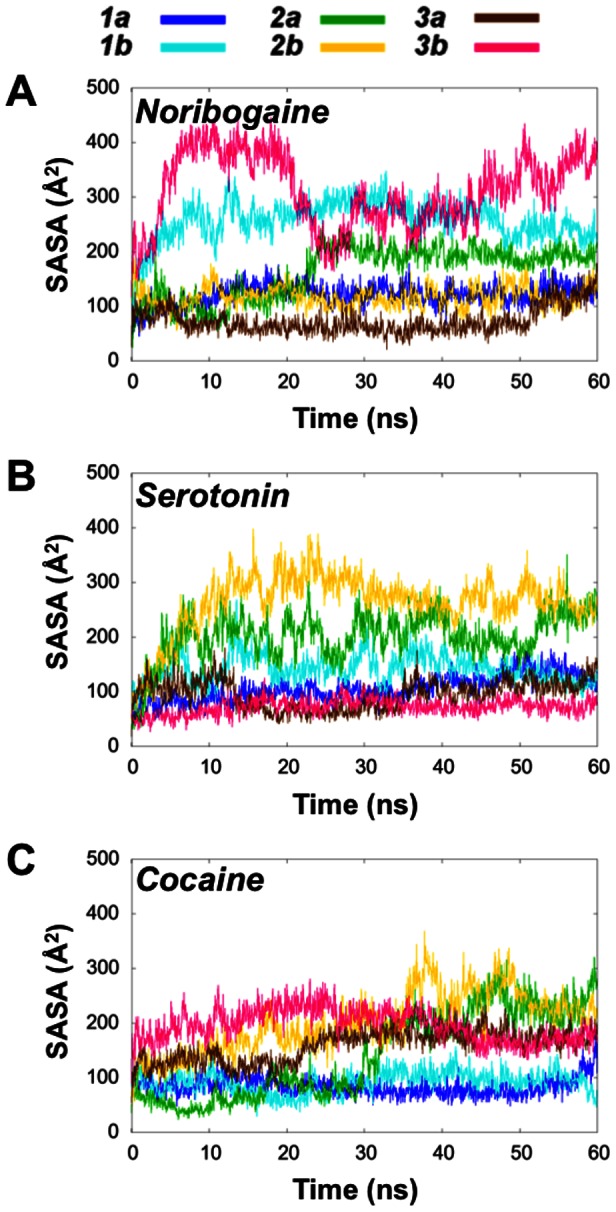
Solvent accessibility of the intracellular pathway residues in hSERT. **A**)–**C**)**.** Plots of the total SASA of the intracellular pathway residues [3], [34], [70] from the noribogaine, serotonin and cocaine systems, respectively.

Similarly, the SASA of the cytoplasmic pathway was measured as an indication of the degree of inward-facing conformation of hSERT formed during the simulations (Figure 4). The cytoplasmic pathway residues were taken as the ones previously determined from experiments to line the putative cytoplasmic pathway [3], [34], [70]: Phe88 (TM1), Phe91 (TM1), Gly94 (TM1), Gly273 (TM5), Ser277 (TM5), Val281 (TM5), Thr284 (TM5), Phe347 (TM6), Ala441 (TM8), Glu444 (TM8), Thr448 (TM8), Ala452 (TM8). This metric has previously been observed to be appropriate in disclosing the formation of the inward-facing transporter conformation [7], [58]. The results reported in Figure 4 reflect the variation in measured SASA of these residues lining the putative pathway from the central binding site to the cytoplasm. It is evident that the noribogaine simulation ***N3b*** reaches the highest SASA value of all systems of about 400 Å^2^. Also the ***N1b***, ***S2a*** and ***S2b*** systems obtain SASA values above 300 Å^2^ for the residues in the internal pathway during the simulations. Two of the cocaine simulations also reach a SASA level of around 300 Å^2^ for short periods of. This increase in SASA may be a result of local rearrangements occurring at a shorter time-scale than opening of the internal pathway since the internal gate is not observed to open as much as in the other two systems. Furthermore, the cocaine-bound system does not sample the same conformational state as the inward-facing LeuT structure as will be discussed below.

**Figure 5:**
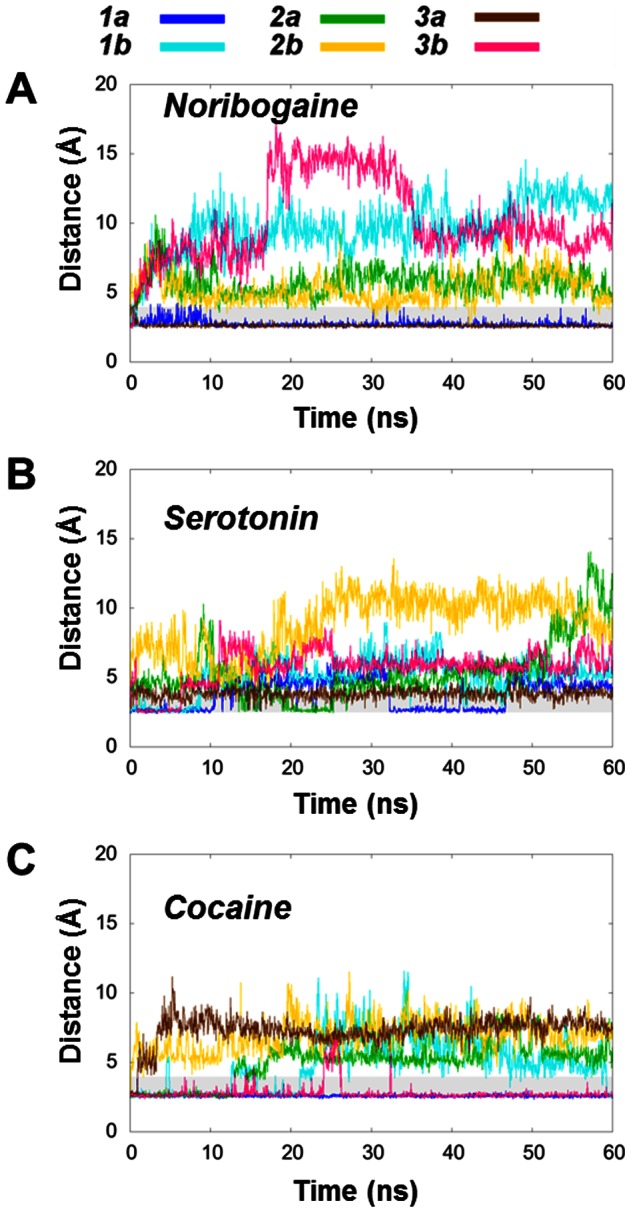
Measurements of the intracellular gating network in hSERT with noribogaine (A), serotonin (B) and cocaine (C). **A**)–**C**)**.** Plot of the shortest distance between the oxygen atom of the hydroxyl of Tyr350 and the carboxylate oxygen atoms in Glu444 as it evolves during the simulations. The grey box highlights the area from 2.5–4 Å.

Motivated by the observed cytoplasmic opening in the serotonin and noribogaine systems, we wanted to clarify the behavior of the intracellular gate (Figure 5). Based on the distance between Tyr350 and Glu444, it is evident that the systems ***N3b***, ***N1b*** and ***S2b*** here show greater extent of opening of some parts of the cytoplasmic pathway than any of the other systems. Again, the data for ***S2a*** also indicate that open conformations are being sampled, though only at the very end of the 60 ns simulation time. The remaining systems all retain a stable interaction similar to what was observed for the ionic interaction in the extracellular vestibule. All of the cocaine systems either remain in a very tight interaction or plateaus at a value around 5–7 Å, which, given the stable nature of the interaction, could indicate rearrangement of interaction partners. Some of the noribogaine and serotonin systems also equilibrate to a distance around 5 Å, though not nearly as open as found in the four most open simulations showing distances of around 10 Å.

**Figure 6:**
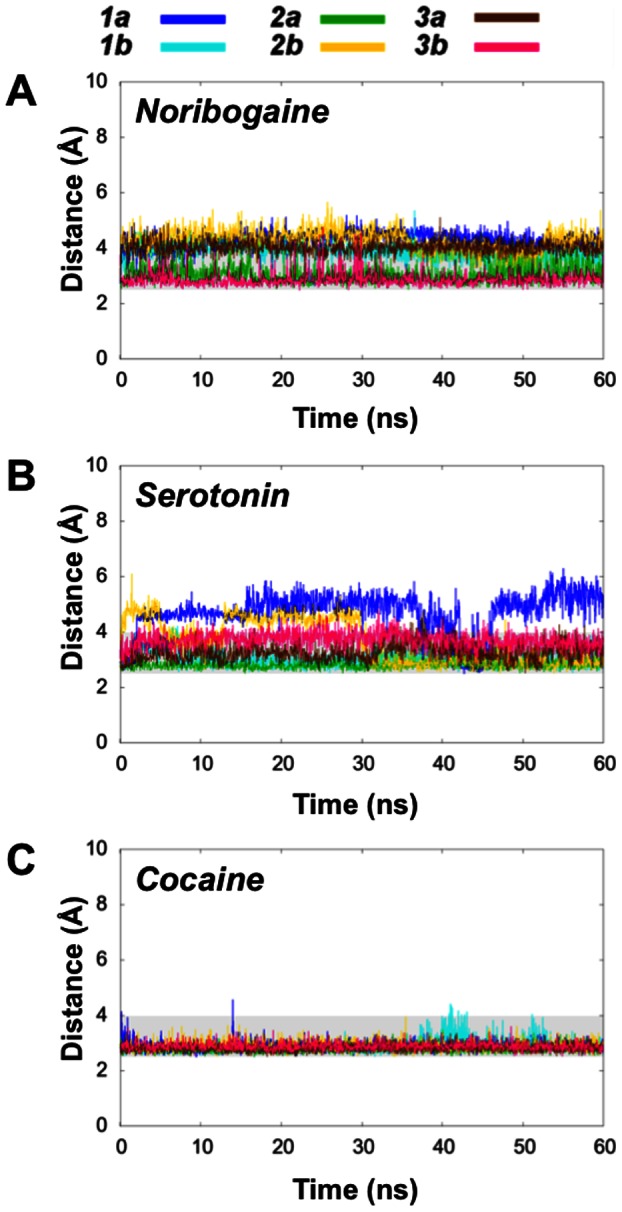
Stability of the Glu136-Gly340 interaction network in hSERT with noribogaine (A), serotonin (B) and cocaine (C). **A**)–**C**). Plots of the shortest distance between the side chain carboxylate group of Glu136 and the backbone nitrogen in Gly340 during the simulation. The grey shadow spans the region between 2.5 Å and 4 Å.

A conserved network around a Glu-Glu pair within hSERT has been proposed to play a role in controlling the transport mechanism [71]. This Glu-Glu pair consisting of Glu136 (TM2) and Glu508 (TM10) is located adjacent to the unwound part of TM6. Interactions involving these residues could therefore play a role in controlling the stability of scaffold-bundle movements. In all the starting structures for the MD simulations an interaction between Glu136 (TM2) and the backbone amide of Gly340 (TM6) was present. The stability of this interaction could give an indication of whether the bundle part moves in a concerted manner or if the four helices within the bundle are able to move independently. A breaking of the Glu136-Gly340 interaction allows for movement of TM2 and TM6 with respect to each other and thus may indicate that the four-helix bundle does not move in a fully concerted manner. The interaction between the Glu508-Glu136 remains stable in all three systems during the entire trajectory (Figure S4). Other specific interactions are present linking the scaffold and bundle, as was shown in Figure 1. The interaction distance between the Glu136-Gly340 linking TM1 and TM6 within the bundle is depicted in Figure 6. It seems to be a trend that the noribogaine and serotonin systems, which are expected to open to the intracellular space, show a larger separation between these two residues, while the interaction is very stable in all cocaine systems during the simulation. Binding of the competitive inhibitor cocaine is observed to stabilize the interactions between the bundle and scaffold hereby preventing the inward-facing transition, whereas the substrate and noribogaine show tendencies towards a weakening of some of the interactions keeping the bundle and scaffold together, hereby allowing a movement of the bundle.

**Figure 7:**
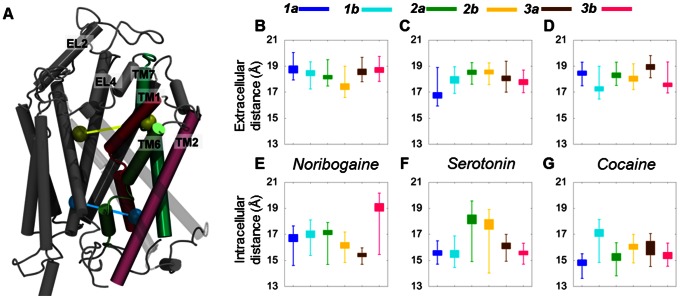
Movement of the bundle with respect to the scaffold. **A**) The scaffold together with TM11 and TM12 are shown in grey. TM10 and TM11 are transparent for clarity. TM1 (dark red), TM2 (pink), TM6 (green) and TM7 (green) correspond to the bundle in hSERT. The extracellular part of the scaffold was defined as the center of mass of the C_α_ atoms of the third and fourth extracellular residues of TM3, TM4, TM5, TM8, TM9, and TM10 at the extracellular side, with the bundle part defined similarly as the center of mass of the C_α_ atoms in the fourth and third extracellular residues of TM1, TM2, TM6, and TM7. These two centers of mass for the bundle and scaffold are shown as yellow balls connected by a line. The intracellular scaffold-bundle distance is similarly defined from the corresponding intracellular residues and the intracellular scaffold-bundle distance is illustrated by cyan spheres connected by a line. **B**) –**D**) Boxplots of the extracellular scaffold-bundle distance. The region corresponding to 50% of the data points is shown as a box and the minimum and maximum values are represented by whiskers. **E**) –**G**) Boxplots of the intracellular scaffold-bundle distance.

In the attempt of identifying a collective variable describing the conformational changes sampled in the simulations, we turned to a quantitative analysis of the relative movement of the bundle with respect to the scaffold was assessed from the simulations. The extracellular movement was defined as the difference in the distance between the extracellular constituents of the scaffold and the bundle. The extracellular part of the scaffold was defined as the center of mass of the C_α_ atoms of the third and fourth last residues of the C-terminal part of TM3, TM5 and TM9 and the N-terminal parts of TM4, TM8 and TM10. The third and fourth residues were chosen to avoid unnatural effects caused by α-helix unwinding at the extracellular helix end. The extracellular part of the bundle was likewise defined as the center of mass of the C_α_ atoms of the third and fourth most extracellular residues, namely of the C-terminal of TM1b and TM7 and the N-terminal of TM2 and TM6a. The distance between the extracellular part of the scaffold and the bundle within the starting structure of the noribogaine simulations is illustrated as a yellow line in Figure 7A. The intracellular distance was defined in a similar manner; the intracellular part of the scaffold consists of the N-terminal of TM3, TM5, and TM9 in addition to the C-terminal of TM4, TM8 and TM10, while the intracellular part of the bundle was defined from the N-terminal of TM1a and TM7 and the C-terminal of TM2 and TM6b. An example of this distance can also be seen in Figure 7A (in cyan).

As judged from boxplots of the extracellular scaffold-bundle distance observed in Figure 7B–D all three types of systems remain relatively stable, and there are no large differences between the three systems. Such stabilization of the extracellular gate upon ligand binding has been observed from EPR-experiments [69] as well as MD simulations [7]. As opposed to this behavior, the intracellular scaffold-bundle distances (Figure 7E–G) differ between the systems. In the ***N3b***, ***S2a*** and ***S2b*** systems, where the SASA of the residues in the internal pathway was observed to reach above 300 Å^2^, the intracellular distance between scaffold and the bundle increases with as much as 5 Å. An increase in the intracellular distance is also observed in the ***C1b*** simulation, however since no substantial increase was observed in the SASA of the intracellular pathway, this is most likely an indication of small rearrangements of the intracellular parts. Generally, greater intracellular separations are found in the noribogaine and serotonin systems compared to the cocaine systems, in which the distances decrease indicating a slightly closing movement of the intracellular bundle-scaffold interface. This behavior suggests that the bundle does not move as a rigid body with respect to the scaffold since larger deviations are observed on the intracellular side of the bundle than on the extracellular side, similar to earlier reports [7], and to what can be inferred from the inward-facing conformation of LeuT where a similar distance measured in the extracellular parts only decrease by 1.5 Å while the intracellular distance increases by 5 Å between the outward-occluded state (PDB code: 2A65 [5]) and the inward-facing conformation (PDB code: 3TT3 [25]).

**Figure 8:**
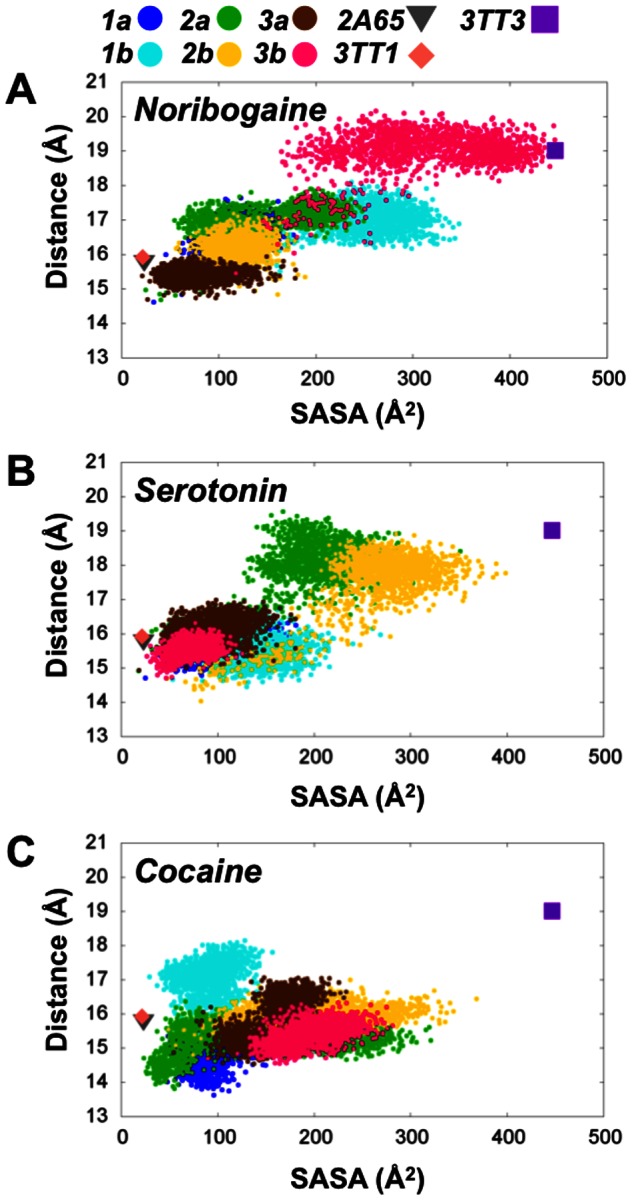
Correlation between SASA of the intracellular pathway and the intracellular distance between the scaffold and bundle. The intracellular scaffold-bundle distance is plotted as a function of the calculated SASA of the intracellular pathway for **A**) noribogaine, **B**) serotonin and **C**) cocaine respectively. In all plots the LeuT crystal structures have been included as reference points; PDB code 2A65 [5] in grey, PDB code 3TT1 [25] as pale red, and PDB code 3TT3 [25] in purple.

The existence of a correlation between the calculated SASA of the intracellular pathway and the intracellular movements of the bundle with respect to the scaffold was further explored and compared to available LeuT crystal structures. In Figure 8 the two variables are plotted and the data for three LeuT crystal structures are included, representing the outward-open (PDB code: 3TT1 [25]), outward-occluded (PDB code: 2A65 [5]) and the inward-open (PDB code: 3TT3 [25]) conformations of LeuT. This comparison clearly indicates that only the noribogaine system is able to reach a conformational state similar to the inward-facing LeuT crystal structure (PDB code: 3TT3 [25]), see Figure 8A. The serotonin systems sample intermediate conformations and do not reach a fully inward-facing conformation, as seen for the inward-facing LeuT structure. Since the latter was solved with four mutations and in complex with a Fab-domain, the conformation seen might be slightly perturbed compared to wild-type LeuT and hSERT (Figure 8B). The cocaine system only samples a small area of the conformational space similar to the outward-facing and outward-occluded LeuT crystal structures (PDB code 3TT1 [25] and 2A65 [5] respectively), see Figure 8C. Although, some of the cocaine systems reached relatively high SASAs for short periods of time (Figure 4), this is never observed in concert with an opening of the internal gate.

Full or partial release of the sodium ion residing in the Na2-site is observed during the transition to the inward-facing conformation of the transporter, similarly to reported behavior for hSERT and LeuT [7], [72]. In two of the noribogaine and three of the serotonin simulations this sodium ion is displaced by up to 6 Å towards the intracellular side, and it becomes fully released in the ***S1a*** system. A minor displacement is also observed in the ***C2b*** system. The other sodium ion, in the Na1 site, and the chloride ion both remain stable in all systems (Figure S5). This suggests that one of the events involved in the translocation of hSERT from an outward-facing conformation to an inward-facing conformation is associated with release of Na2 to the cytoplasm.

**Figure 9:**
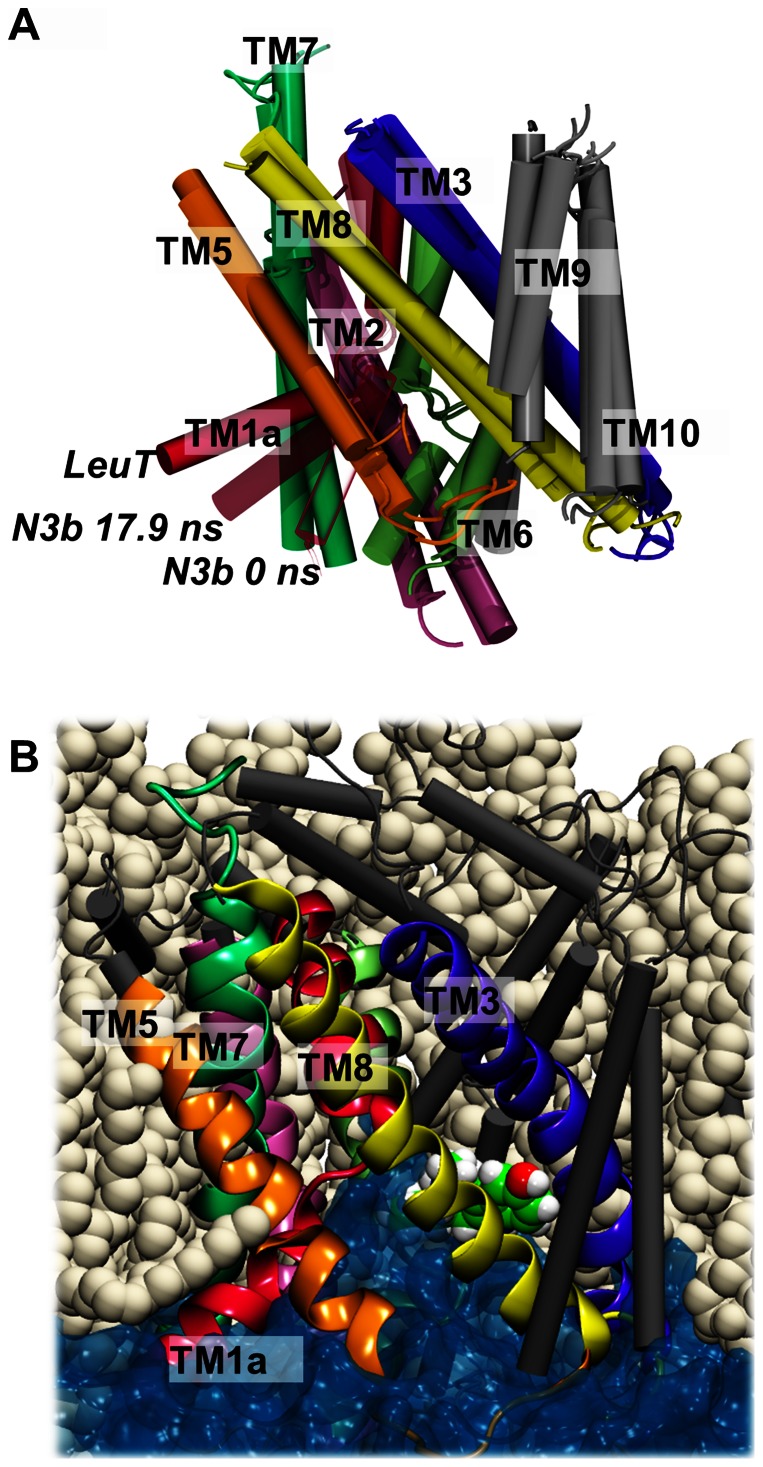
Similarities in conformational changes in LeuT and hSERT. **A**) The inward-facing crystal structure of LeuT compared to the inward-facing conformation sampled in the noribogaine MD simulations as well as the initial outward occluded conformation of hSERT. It is evident that TM1a is responsible for the largest movements during the transition towards the inward-facing conformation. **B**) The stabilization of the inward-facing conformation of the noribogaine system is accomplishes by mutual water and lipid adaption.

In the recently published inward-facing structure of, LeuT [25], the protein contained four mutations in addition to a Fab-domain bound at the intracellular side of the protein to ease crystallization of the transporter. The structure revealed the movement of TM1a as the dominant movement of the TM domain compared to the outward-facing conformation of LeuT. The tilt angle of TM1a with respect to the scaffold was monitored for all three systems during the simulations (Figure S6). An outward movement of TM1a in ***N3b*** is observed, similar to what was observed in the inward-facing conformation of LeuT [25]. In contrast, TM1a is observed to close towards the scaffold in two of the cocaine systems (***C1b*** and ***C2a***), and also in ***N2a***. The most inward-facing snapshot of hSERT with bound noribogaine extracted from the MD simulations was used for comparison with this inward-facing structure of LeuT. The most inward-facing snapshot was selected as occurring at the time step where the SASA of the internal pathway residues is highest (in ***N3b*** after approximately 20 ns). By aligning the scaffold of LeuT and hSERT it becomes evident that the conformational changes observed during the transition to the inward-facing conformation results in a conformation of hSERT that is similar to the LeuT crystal structure (Figure 9), with a calculated RMSD of 2.92 Å between the bundle in ***N3b*** and the inward-facing LeuT (PDB code 3TT3 [25]). Since TM1 is located on the outer surface of the transporters the adaption of the water and lipid environment to the changed protein conformation was analyzed further. It was found that water molecules enter the cytoplasmic pathway of the transporter as the protein conformation changed towards an inward-facing conformation. Prior to the simulation, the cytoplasmic pathway of the transporter was tightly packed with protein and completely desolvated. The lipid bilayer surrounding the transporter dimer additionally aid in the stabilization of the inward-facing conformation by packing around TM1a. In Figure 9B it can be seen how a lipid molecule is lining TM1a. The lipids and protein were accordingly able to mutually adapt to each other and the relatively large conformational change observed for TM1a did not result in any unphysical movements of the helix tip into the hydrophobic part of lipid bilayer. The similarity between the conformations sampled by the noribogaine bound transporter and inward-facing LeuT is also evident from Figure 8, where an overlap in the conformational state defined by the solvent accessibility of the intracellular pathway and the scaffold-bundle distance on the intracellular site is seen.

**Figure 10:**
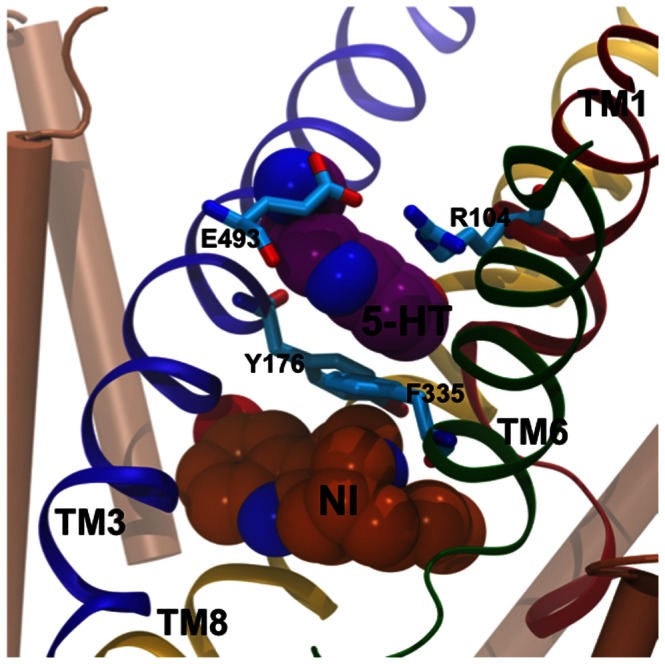
Binding of serotonin to the extracellular binding pocket of hSERT. A representative pose of serotonin binding in the extracellular binding site as obtained from induced fit docking. Serotonin is shown in purple spheres, and noribogaine (in the central binding pocket) is shown in orange spheres. TM1 (red), TM3 (blue), TM6 (green) and TM8 (yellow) are shown in cartoon. The amino acid residues in the extracellular gate are shown in orange sticks. TM2, TM4, TM5, TM7 and TM9 are shown as beige cylinders.

For LeuT and the dopamine transporter, a two substrate transport mechanism has been suggested [73], [74]. In this mechanism the binding of a second substrate in an extracellular site, known as the S2 site, facilitates the conformational change from outward- to inward-facing. If such a mechanism is valid for hSERT, an additional extracellular cavity in the inward-facing conformation must be present. This cavity should be large enough to accommodate the substrate. To investigate this, we chose the most inward-facing conformation from the serotonin and noribogaine simulations, respectively, and performed IFD of serotonin in the suggested S2 site. The hSERT structures from the simulations were chosen based on achieving the maximum SASA value of the residues lining the intracellular pathway, and the structures were extracted after 15.7 ns for the ***S2b*** serotonin system, while the structure obtained at 17.9 ns from ***N3b*** was selected as the inward-facing noribogaine system. For both hSERT conformations, several poses with serotonin in the extracellular cavity were obtained (please see the details in Supporting Information S1). The poses of serotonin in the S2 site within the ***N3b*** system form distinct clusters with the ligand in the largest cluster placed in a site similar to the S2 site suggested for LeuT [73] as shown in Figure 10. The poses of serotonin within the S2 site in the inward-facing hSERT extracted from the ***S2b*** MD simulation cannot easily be clustered as they seem to be randomly distributed over a rather broad volume of the extracellular cavity, positioned around the proposed S2 site. The hSERT structure extracted from the ***N3b*** system, representing the inward-facing noribogaine system is considerably more inward-facing than the snapshot structure from ***S2b*** serotonin system. The docking results thus suggest that the extracellular cavity is more enclosed in the inward-facing noribogaine structure confining the space available for a second serotonin to occupy. Since it is possible to observe the conformational change from an outward- to inward-facing conformation during the simulations without a substrate in the extracellular site, the results here indicate that a second substrate may not be a necessity for transport to occur. However, this does not exclude the possibility that the transport rate could be enhanced when a second substrate binds to the transporter in the S2 site, hereby ensuring that a new substrate is ready for transport as soon as the transporter returns to an outward-facing conformation. Loland and co-workers recently suggested that the extracellular cavity in hSERT is capable of binding small molecular ligands and that known antidepressant such as (*S*)-citalopram and clomipramine bind in an allosteric fashion in this site [75]. Thus it seems that S2 site is indeed relevant for hSERT, at least in the context of transport inhibitors.

## Conclusion

**Figure 11:**
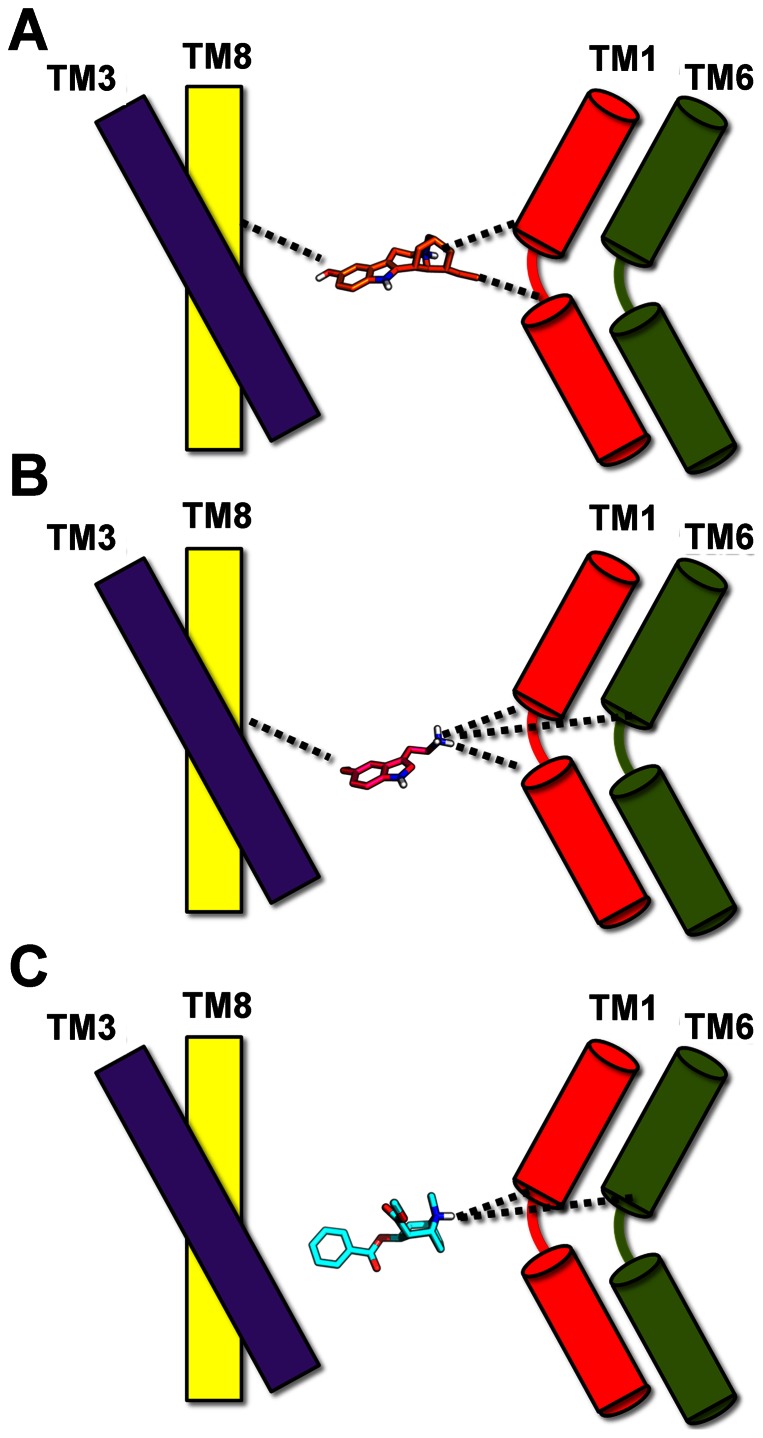
Ligand dependent bundle-scaffold interactions. **A**) Noribogaine accommodates interactions between TM1 of the bundle (Asp98 (TM1) and Tyr 95 (TM1)) and the scaffold either through Ser438 (TM8) or Ala169 (TM3). **B**) Serotonin maintains stable interactions between the scaffold (Ser438 (TM8)) and both TM1 and TM6 (Asp98 (TM1), Tyr95 (TM1) and Phe335 (TM6)) in the bundle. **C**) Cocaine only interacts with TM1 (Asp98) and TM6 (Phe335) of the bundle.

The MD simulations presented here capture ligand induced behaviour of hSERT as observed from experiments and provide atomic level information related to the observed conformational changes. We observe that cocaine retains hSERT in an outward-facing conformation, while noribogaine, and to some extend the substrate, is able to induce the transition to an inward-facing state at the time-scale sampled. The conformational changes during the transition to the inward-facing state reveal that the largest movement is found in TM1a, which is similar to what was recently observed in the first high resolution crystal structure of an inward-facing LeuT structure [25]. It is also evident that the transitions observed in this study utilizing the OPLS_2005 force field [46], [54] captures the same motions as seen in a recent study of substrate bound hSERT [7] utilizing the CHARMM force field [56]. The main question however still is how these three types of ligands can induce different conformational changes of hSERT. One of the major differences in the molecular composition of noribogaine, serotonin and cocaine is that the latter does not contain a functional group that enables it to bridge the scaffold and bundle through selective electrostatic interactions. It has previously been suggested that the hydroxyl group of serotonin is important for substrate binding [12]. The interactions sampled between noribogaine and the protein during the simulations reveal that the ligand bridges the scaffold and the bundle through stable interactions with TM1 in the bundle (Asp98 and Tyr95) and with either TM8 or TM3 within the scaffold (Ser438, Gly442 or Ala169, respectively) (Figure 11). The binding of serotonin to hSERT results in a similar interaction network being established between the scaffold and bundle; however serotonin induces the formation of an additional stable interaction with TM6 of the bundle (Phe335), as seen in Figure 11. The competitive inhibitor cocaine, which stabilizes an outward-facing protein conformation, however differs from the two inward-facing inducing ligands with respect to establishing an interaction network between the bundle and scaffold. Since cocaine lacks a functional group able to form selective electrostatic interactions with the scaffold, this ligand does not bridge the scaffold and bundle in the same way as serotonin and noribogaine do. As a result cocaine only forms stable polar interactions with the bundle though Asp98 (TM1) and Phe335 (TM6). It can therefore be speculated that a ligand dependent interplay between the bundle and scaffold is required and that the bound ligand must be able to interact with both protein parts through selective electrostatic interactions in order to induce the required movements for the conformational change to occur from an outward- to inward-facing structure. A possible rationale for transport of serotonin but not noribogaine by hSERT, could then be that serotonin is smaller than noribogaine, with consequently larger movements of the TM parts being required for transport of the latter. The smaller size of the substrate would additionally enable the ligand to diffuse easier from the central binding pocket compared to a larger molecule. Another factor possibly required for substrate translocation could also be the movement of TM6 with which only the substrate and not the non-competitive inhibitor interacts with. The tendency of noribogaine to stabilize an inward-facing conformation of hSERT may suggest a binding pathway of this compound from the cytoplasm. It can be speculated that noribogaine, being more hydrophobic than serotonin, may be passively transported into cells similarly to what has been observed for serotonin [74], hereby allowing for binding to hSERT from the intracellular site when in an inward-facing conformation entailing the non-competitive nature of inhibition. Further studies are underway addressing such issues, and we foresee that this knowledge may provide important input for future drug design.

## Supporting Information

Figure S1 **Comparison of the central binding pockets.** TM1(red), TM3(violet), TM6(green) and TM8(yellow) are shown as cartoon. Residue 171 to 174 of TM3 have been omitted for clarity on the left figure, while residues 95 to 101 (TM1) and 437 to 443 (TM8) have been omitted for clarity on the right figure. The residues found within 5 Å of 5-HT are shown as side chains for all three protein-ligand complexes in orange (noribogaine), purple (serotonin) and cyan (cocaine). The volume the ligands occupy have been illustrated by transparent surfaces with noribogaine in orange, serotonin in purple and cocaine in cyan.
(TIF)

Figure S2 **Stability of the systems with the three ligands; noribogain (left column), 5-HT (middle column) and cocaine (right column) during the MD simulations.** **A–C** RMSD of C_α_ atoms in each monomer relative to the strating structure. **D–F**. RMSD of C_α_ atoms in the TM part of the monomers relative to the starting structure. **G–I** RMSF of the C_α_ atoms in the monomers aligned according to the full dimer. **J–L**. RMSD of all non-hydrogen atoms in the ligands relative to the initial frame aligned according to the C_α_ atoms in the TM parts of the protein.
(TIF)

Figure S3 **Dynamics of the extracellular salt bridge formed by Arg104 and Glu493 in hSERT.** A. The extracellular lid is composed by residues Arg104, Tyr176, Phe335 and Glu493, which are all displayed as grey sticks, while the helix they belong to is represented in cartoon. Arg104 and Glu493 constitute the extracellular salt bridge, which is formed when the transporter is fully or partial closed towards the extracellular environment. The aromatic lid is composed by Tyr176 and Phe335 and also blocks the extracellular pathway when the transporter is closed to the outside. TM8 is shown in the background as transparent and noribogaine (orange sticks) below the lid, both for orientation B-D Plots of the shortest distance between the nitrogen atoms in the guanidinum group of Arg104 and the carboxylate group in Glu493(OE) as measured during the simulations with noribogaine, 5-HT and cocaine. The grey shadow spans from the lower border of a typical hydrogen bond or charged hydrogen bond (2.5 Å) and the upper boarder of a salt bridge (4 Å).
(TIF)

Figure S4 **Measurements of the intracellular gating network in hSERT with noribogaine A), serotonin B) and cocaine C**)**.** Plots of the distance between the side chain interaction between Glu136 (OE) and the protonated oxygen in the side chain of Glu508 (OH). The grey shadow spans from highlights the 2.5–4 Å area.
(TIF)

Figure S5 **Ion z-displacement from initial position within the three systems.** Na1 z-displacement in the **A**) noribogaine, **B**) serotoin and **C**) cocaine system. Na2 z-displacement in the **D**) noribogaine, **E**) serotoin and **F**) cocaine system. Cl^−^ z-displacement in the **G**) noribogaine, **H**) serotoin and **I**) cocaine system.
(TIF)

Figure S6 **Kink of TM1a with respect to the scaffold for all three systems.** TM1a kink in the **A**) noribogaine, **B**) serotonin and **C**) cocaine system.
(TIFF)

Supporting Information S1 **Further information about docking simulations and analysis as well as details of the MD simulations.**
(DOCX)

Table S1 **Noribogaine IFD data in hSERT.** The data is arranged according to the binding modes and provides an overview of selected distances between the three hetero atoms in noribogaine (N+ and OH) and hetero atoms in amino acid residues within the binding site pocket as well as the GlideScore, Emodel, and IFDScore for each pose. The representative pose of each binding mode is marked with a grey shadow. The RMSD given is between the binding mode representative (gray shadow) and the current pose. For the outliers the RMSD is relative to the representative of **N**–**I**.
(DOCX)

Table S2 **Cocaine IFD data.** The data is arranged according to the binding modes or clusters and provides an overview of selected distances between the quaternary ammonium in cocaine (N^+^) and selected hetero atoms in amino acid residues within the binding site pocket as well as the GlideScore, Emodel, and IFDScore for each pose. The representative pose of **C**–**I** is marked with a grey shadow. The RMSD given is between the binding mode representative and the current pose. For the outliers the RMSD is relative to the representative of **C**–**I**.
(DOCX)
