# Supplementary figures and images for: Ligand Induced Conformational Changes of the Human Serotonin Transporter Revealed by Molecular Dynamics Simulations
Source: PLoS One. 2013 Jun 12;8(6):e63635. doi: 10.1371/journal.pone.0063635 (PMC3680404; doi:10.1371/journal.pone.0063635)

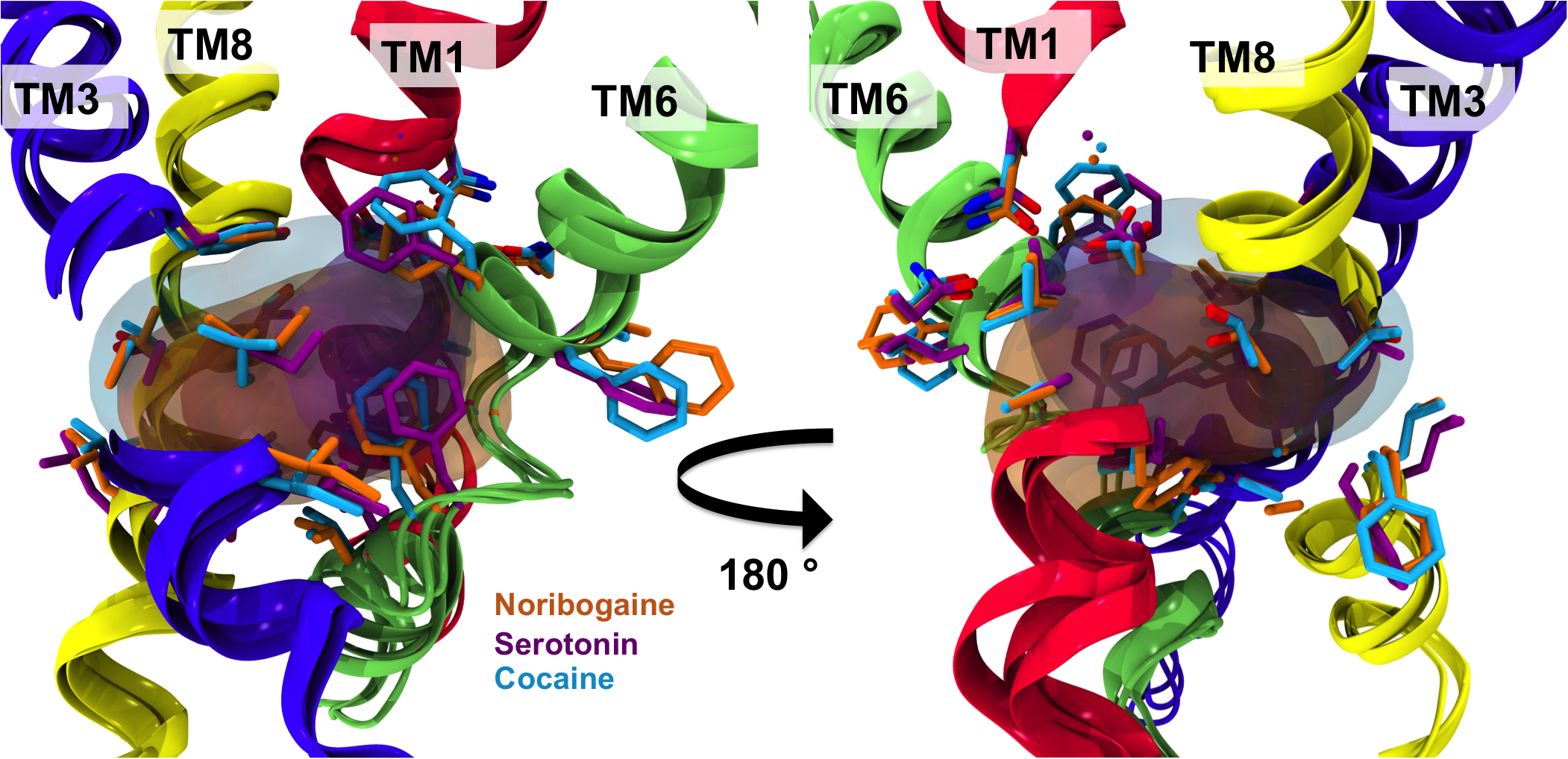

Supplement: Figure S1 — Comparison of the central binding pockets. TM1(red), TM3(violet), TM6(green) and TM8(yellow) are shown as cartoon. Residue 171 to 174 of TM3 have been omitted for clarity on the left figure, while residues 95 to 101 (TM1) and 437 to 443 (TM8) have been omitted for clarity on the right figure. The residues found within 5 Å of 5-HT are shown as side chains for all three protein-ligand complexes in orange (noribogaine), purple (serotonin) and cyan (cocaine). The volume the ligands occupy have been illustrated by transparent surfaces with noribogaine in orange, serotonin in purple and cocaine in cyan. (TIF) [file pone.0063635.s001.tif]

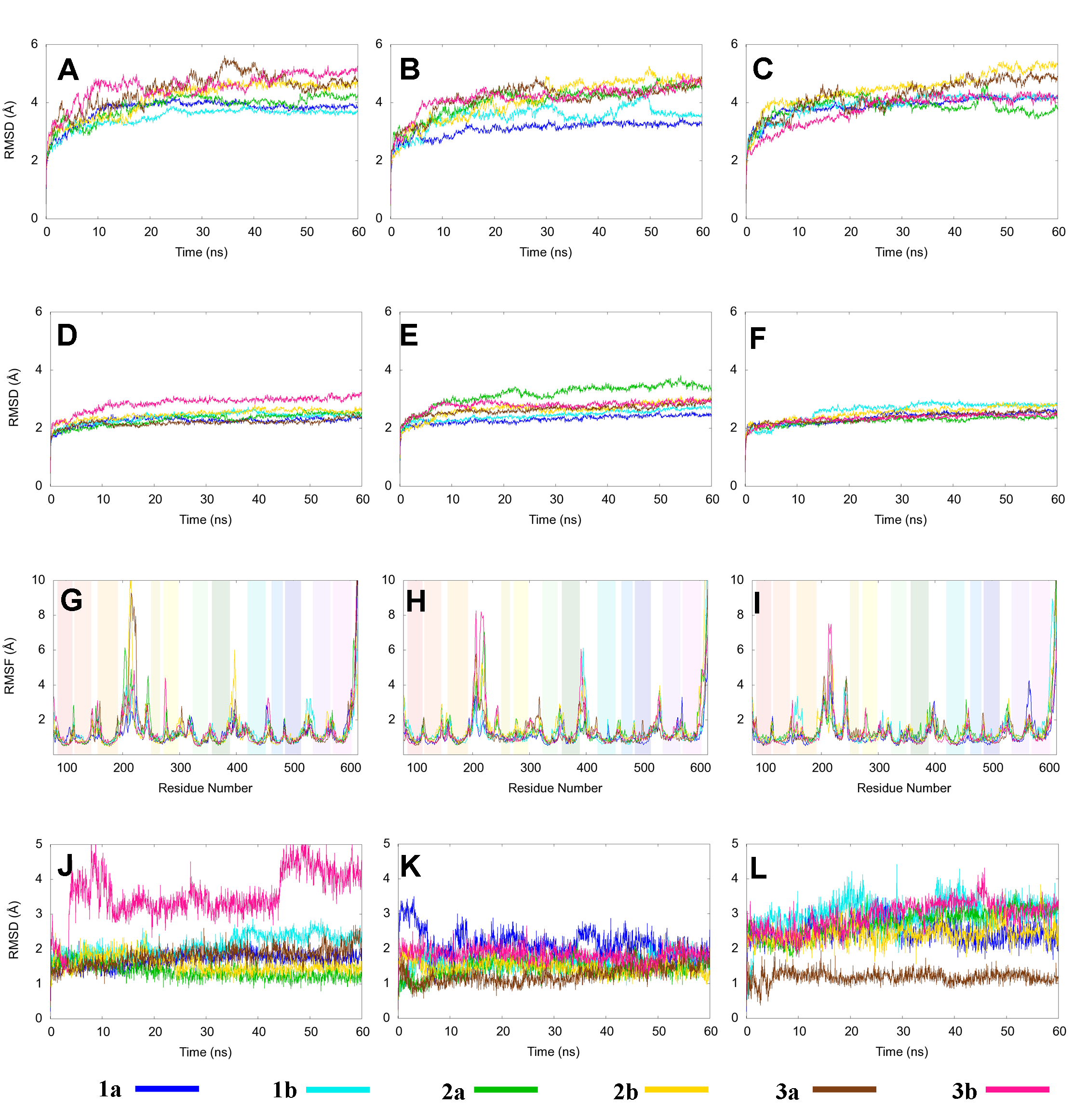

Supplement: Figure S2 — Stability of the systems with the three ligands; noribogain (left column), 5-HT (middle column) and cocaine (right column) during the MD simulations. A–C RMSD of Cα atoms in each monomer relative to the strating structure. D–F. RMSD of Cα atoms in the TM part of the monomers relative to the starting structure. G–I RMSF of the Cα atoms in the monomers aligned according to the full dimer. J–L. RMSD of all non-hydrogen atoms in the ligands relative to the initial frame aligned according to the Cα atoms in the TM parts of the protein. (TIF) [file pone.0063635.s002.tif]

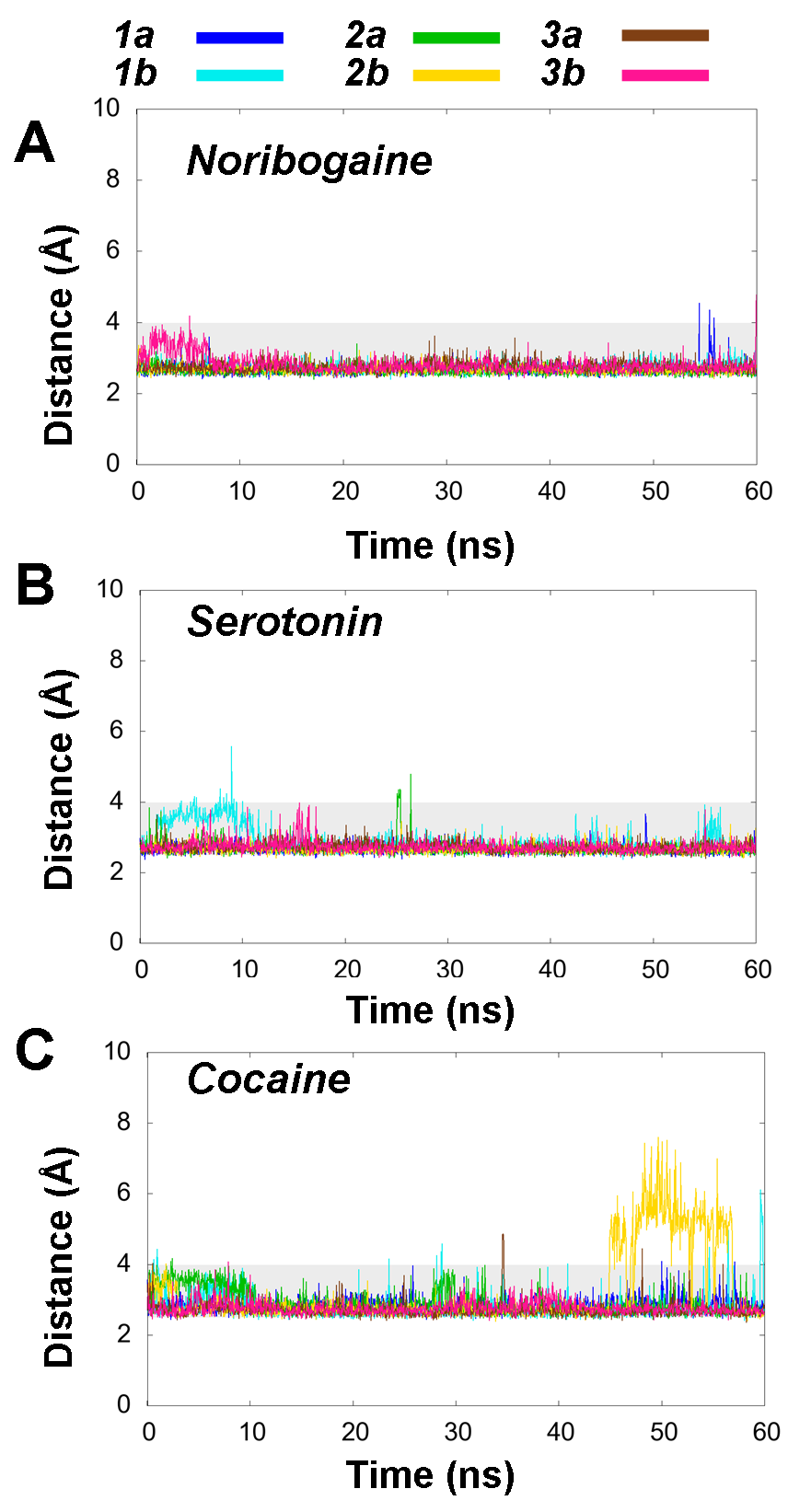

Supplement: Figure S3 — Dynamics of the extracellular salt bridge formed by Arg104 and Glu493 in hSERT. A. The extracellular lid is composed by residues Arg104, Tyr176, Phe335 and Glu493, which are all displayed as grey sticks, while the helix they belong to is represented in cartoon. Arg104 and Glu493 constitute the extracellular salt bridge, which is formed when the transporter is fully or partial closed towards the extracellular environment. The aromatic lid is composed by Tyr176 and Phe335 and also blocks the extracellular pathway when the transporter is closed to the outside. TM8 is shown in the background as transparent and noribogaine (orange sticks) below the lid, both for orientation B-D Plots of the shortest distance between the nitrogen atoms in the guanidinum group of Arg104 and the carboxylate group in Glu493(OE) as measured during the simulations with noribogaine, 5-HT and cocaine. The grey shadow spans from the lower border of a typical hydrogen bond or charged hydrogen bond (2.5 Å) and the upper boarder of a salt bridge (4 Å). (TIF) [file pone.0063635.s003.tif]

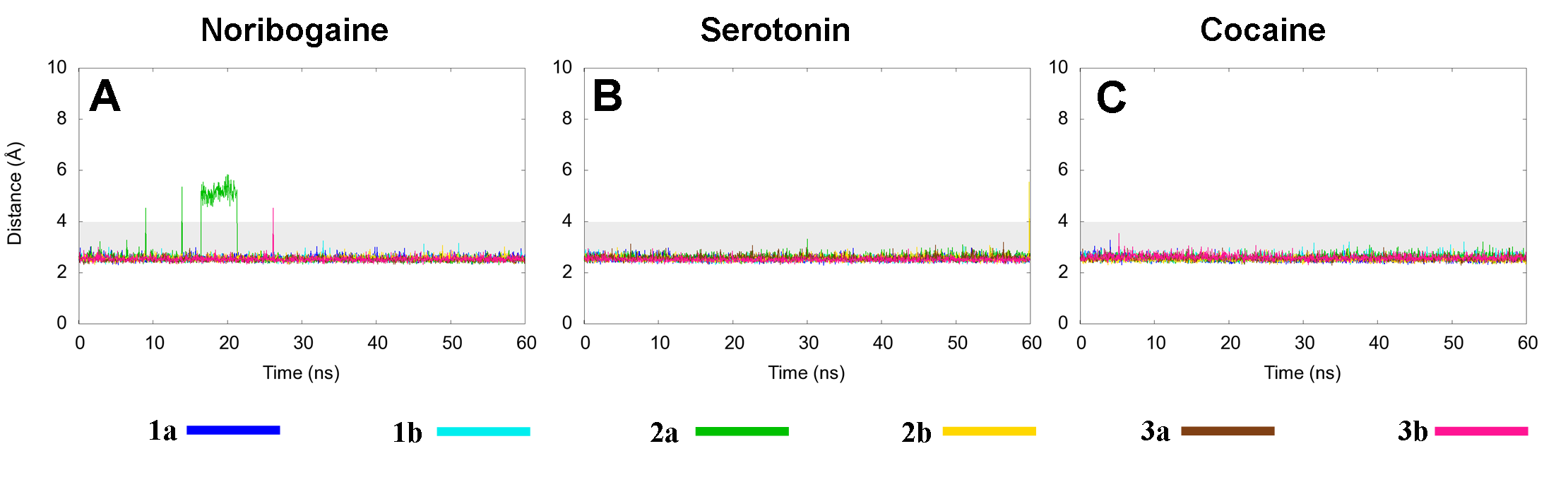

Supplement: Figure S4 — Measurements of the intracellular gating network in hSERT with noribogaine A), serotonin B) and cocaine C). Plots of the distance between the side chain interaction between Glu136 (OE) and the protonated oxygen in the side chain of Glu508 (OH). The grey shadow spans from highlights the 2.5–4 Å area. (TIF) [file pone.0063635.s004.tif]

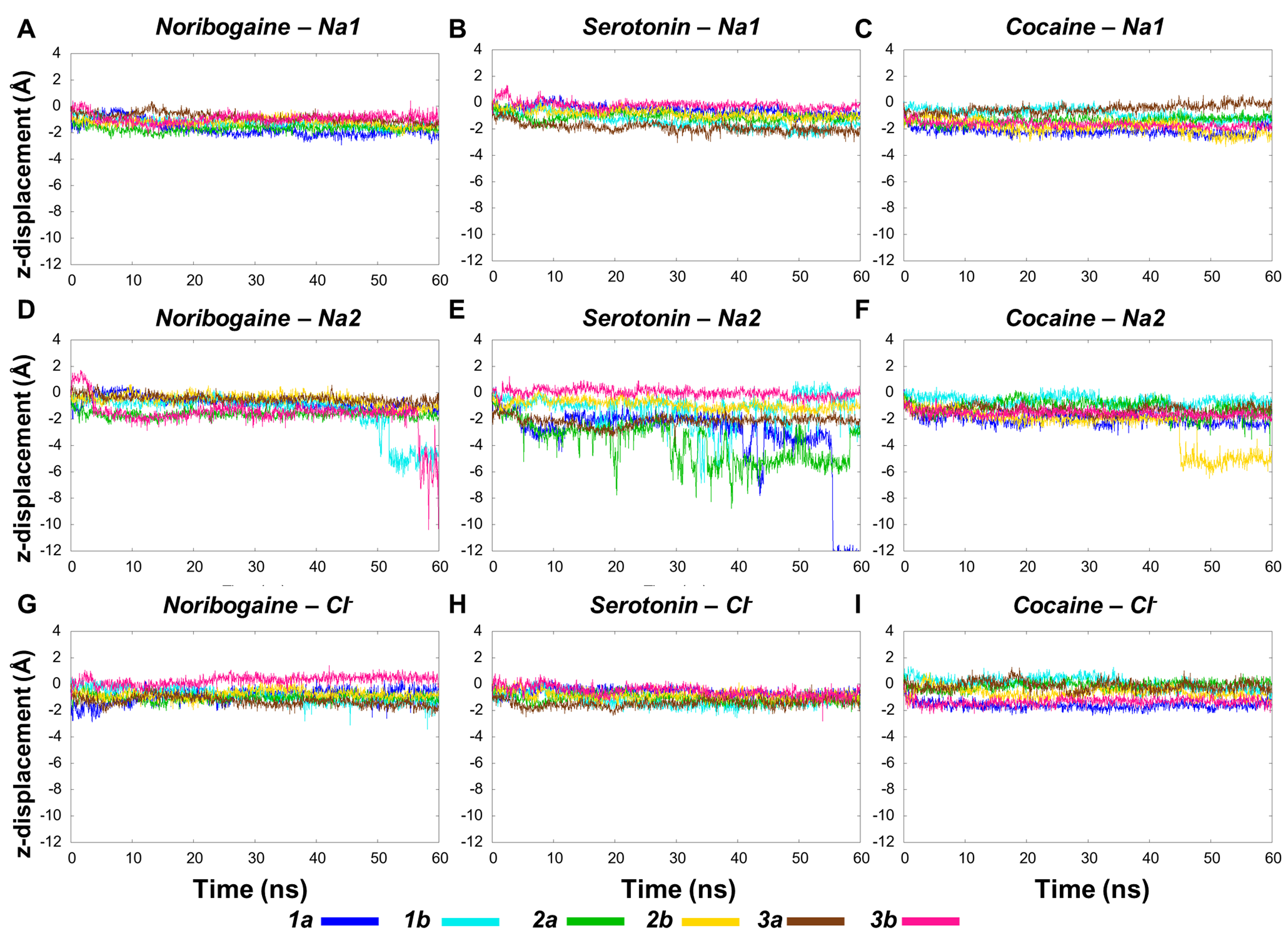

Supplement: Figure S5 — Ion z-displacement from initial position within the three systems. Na1 z-displacement in the A) noribogaine, B) serotoin and C) cocaine system. Na2 z-displacement in the D) noribogaine, E) serotoin and F) cocaine system. Cl− z-displacement in the G) noribogaine, H) serotoin and I) cocaine system. (TIF) [file pone.0063635.s005.tif]

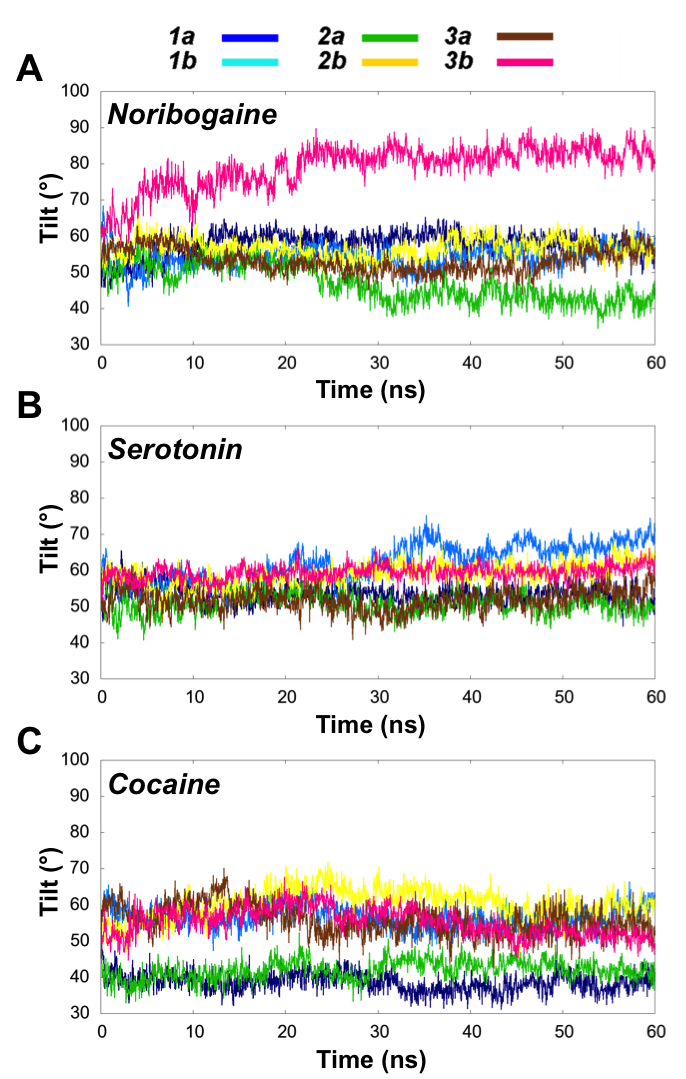

Supplement: Figure S6 — Kink of TM1a with respect to the scaffold for all three systems. TM1a kink in the A) noribogaine, B) serotonin and C) cocaine system. (TIFF) [file pone.0063635.s006.tiff]
