# Supplementary material for: Ligand Induced Conformational Changes of the Human Serotonin Transporter Revealed by Molecular Dynamics Simulations
Source: PLoS One. 2013 Jun 12;8(6):e63635. doi: 10.1371/journal.pone.0063635 (PMC3680404; doi:10.1371/journal.pone.0063635)
Supplement: Supporting Information S1 — Further information about docking simulations and analysis as well as details of the MD simulations. (DOCX) [file pone.0063635.s007.docx]

*Supporting Information for*

**Ligand induced conformational changes of the human serotonin transporter revealed by molecular dynamics simulations**

Heidi Koldsø,^¶1 §#^ Henriette Elisabeth Autzen,^1§^ Julie Grouleff,^§^ and Birgit Schiøtt^¶§*^

The ^¶^Center for Insoluble Protein Structures (*in*SPIN) and the Interdisciplinary Nanoscience Center (*i*NANO), **^§^**Department of Chemistry, Aarhus University, Aarhus, Denmark

*Address correspondence to: Birgit Schiøtt, Phone: +45 8715 5975, Fax: +45 8619 6199 Email: birgit@chem.au.dk

^#^ Current Address: Department of Biochemistry, University of Oxford, Oxford, United Kingdom

^1^These authors contributed equally

**Analysis of IFD poses in S1**

IFD of noribogaine in hSERT yielded fourteen poses, which were clustered according to an RMSD ≤ 2 Å for all non-hydrogen atoms within noribogaine. Noribogaine is fully situated inside the binding site pocket (below the extracellular gate) in six of the poses. One pose was abandoned due to a GlideScore of -7.79 kcal/mol, which compared to the GlideScore of the remaining poses is particularly poor. Based on RMSD the remaining 5 posed were divided into 3 binding modes (**N-I**, **N-II** and **N-III)**. The **N-I** and **N-II** binding mode is similar to the validated serotonin banding mode [1] with the only difference being orientation of the hydroxyl group either towards Ala169 (**N-I**) or Thr439 (**N-II**). Therefore, the lowest energy pose within **N-I** was chosen for the MD simulations both based on probability, similarities to serotonin binding and that this cluster has most favourable combined Gscore and Emodel (Table S1).

Docking of cocaine yielded 19 poses. Nine poses displayed the previously validated binding mode seen in hDAT [2]. The representative pose of cocaine was selected based on the one resembling the validated binding mode seen in hDAT [2]. Table S2 does illustrate that all poses within **C-I** have almost the same GlideScore (< 1.5 kcal/mol difference) and are very close in RMSD so it is believed that the different poses most likely will be sampled readily during MD simulation. The representative from the cocaine docking calculations is highlighted in Table S2.

The input for the serotonin simulations was chosen based on previously validated orientation [1] within an optimized model [3] and the pose having the validated orientation and lowest GlideScore was chosen (see Koldsø *et al.* [3]).

Only minor differences are observed between the side chain orientations within binding pocket of hSERT between representative poses of the three different ligands (Figure S1).

**Docking of serotonin in the S2-site**

The binding site was defined from residues 104, 407, and 493. The poses were clustered using the conformer_cluster.py script within the Schrödinger suite 2011 [4] based on RMSD for heavy atoms in the ligand applying the average linkage methodology. The number of clusters was chosen based on clustering statistics and an RMSD matrix.

**Details of MD simulations.**

The three simulation systems consisted of the hSERT dimer with a ligand present in the central bindingsite of each monomer. The dimer was embedded in a POPC bilayer, and the sysmte was solvated with water molecules and added NaCl to neutralizing the system and to reach a physiological concentration of 0.2 M. The noribogaine systems had a dimension of 96 Å × 134 Å × 119 Å and contained a total of 120637 atoms, including 189 POPC molecules, 25823 TIP3P water molecules, 290 Na^+^ and 308 Cl^-^ ions. The serotonin systems had a dimension of 95 Å × 133 Å × 118 Å and contained a total of 119686 atoms, including 189 POPC molecules, 25522 TIP3P water molecules, 287 Na^+^ and 305 Cl^-^ ions. The cocaine systems had a dimension of 95 Å × 132 Å × 118 Å and contained a total of 120958 atoms, including 191 POPC molecules, 25842 TIP3P water molecules, 291 Na^+^ and 309 Cl^-^ ions.
