# Supplementary material for: Ligand Induced Conformational Changes of the Human Serotonin Transporter Revealed by Molecular Dynamics Simulations
Source: PLoS One. 2013 Jun 12;8(6):e63635. doi: 10.1371/journal.pone.0063635 (PMC3680404; doi:10.1371/journal.pone.0063635)
Supplement: Table S1 — Noribogaine IFD data in hSERT. The data is arranged according to the binding modes and provides an overview of selected distances between the three hetero atoms in noribogaine (N+ and OH) and hetero atoms in amino acid residues within the binding site pocket as well as the GlideScore, Emodel, and IFDScore for each pose. The representative pose of each binding mode is marked with a grey shadow. The RMSD given is between the binding mode representative (gray shadow) and the current pose. For the outliers the RMSD is relative to the representative of N–I. (DOCX) [file pone.0063635.s008.docx]

**Table S1. Noribogaine IFD data in hSERT.** The data is arranged according to the binding modes and provides an overview of selected distances between the three hetero atoms in noribogaine (N+ and OH) and hetero atoms in amino acid residues within the binding site pocket as well as the GlideScore, Emodel, and IFDScore for each pose. The representative pose of each binding mode is marked with a grey shadow. The RMSD given is between the binding mode representative (gray shadow) and the current pose. For the outliers the RMSD is relative to the representative of **N-I**.

| **Cluster** | **N^+^-Tyr95(O) (Å)** | **N^+^-Asp98(OD) (Å)** | **OH--Ala169(O) (Å)** | **OH--Gly442(O) (Å)** | **OH--Thr439(OH) (Å)** | **RMSD**  **(Å)** | **GlideScore**  **(kcal/mol)** | **Emodel**  **(kcal/mol)** | **IFDScore**  **(kcal/mol)** |
| --- | --- | --- | --- | --- | --- | --- | --- | --- | --- |
| N-I | **3.04** | **4.45** | **3.04** | 4.16 | 6.13 | 0.00 | -12.38 | -20.85 | -877.59 |
| N-I | **3.00** | **4.44** | **3.03** | 4.16 | 6.13 | 0.04 | -12.32 | -21.51 | -877.53 |
|  |  |  |  |  |  |  |  |  |  |
| N-II | **4.63** | **4.22** | 5.22 | 5.68 | **2.61** | 0.00 | -10.78 | -58.64 | -876.36 |
| N-II | **4.94** | **4.03** | 7.68 | 8.78 | **3.62** | 1.69 | -9.55 | -46.87 | -874.50 |
|  |  |  |  |  |  |  |  |  |  |
| N-III | 4.94 | **5.43** | **2.56** | 3.64 | 5.08 | 0.00 | -12.44 | -11.26 | -876.71 |
|  |  |  |  |  |  |  |  |  |  |
| OUT | 7.42 | 5.26 | 11.88 | 14.07 | 10.65 | 6.30 | -11.91 | -43.75 | -878.18 |
| OUT | 6.14 | 3.21 | 6.88 | 6.11 | 12.51 | 5.00 | -13.23 | -38.97 | -878.11 |
| OUT | 5.55 | 4.02 | 12.80 | 14.79 | 11.09 | 5.95 | -12.59 | -45.82 | -878.02 |
| OUT | 5.08 | 4.39 | 12.92 | 14.57 | 11.82 | 5.44 | -12.12 | -19.72 | -877.71 |
| OUT | 7.56 | 5.29 | 12.77 | 14.88 | 11.03 | 6.61 | -11.51 | -41.37 | -877.64 |
| OUT | 6.96 | 6.02 | 10.72 | 13.05 | 10.07 | 5.67 | -11.81 | -43.52 | -877.08 |
| OUT | 5.35 | 4.32 | 12.92 | 14.63 | 11.91 | 5.51 | -12.05 | -23.92 | -876.74 |
| OUT | 6.60 | 5.77 | 9.94 | 12.46 | 9.95 | 5.30 | -11.39 | -45.67 | -876.17 |
| OUT | 7.30 | 6.67 | 5.62 | 6.01 | 2.71 | 4.28 | **-7.79** | -49.48 | -872.11 |
|  |  |  |  |  |  |  |  |  |  |
