# Supplementary material for: Ligand Induced Conformational Changes of the Human Serotonin Transporter Revealed by Molecular Dynamics Simulations
Source: PLoS One. 2013 Jun 12;8(6):e63635. doi: 10.1371/journal.pone.0063635 (PMC3680404; doi:10.1371/journal.pone.0063635)
Supplement: Table S2 — Cocaine IFD data. The data is arranged according to the binding modes or clusters and provides an overview of selected distances between the quaternary ammonium in cocaine (N+) and selected hetero atoms in amino acid residues within the binding site pocket as well as the GlideScore, Emodel, and IFDScore for each pose. The representative pose of C–I is marked with a grey shadow. The RMSD given is between the binding mode representative and the current pose. For the outliers the RMSD is relative to the representative of C–I. (DOCX) [file pone.0063635.s009.docx]

**Table S2. Cocaine IFD data.** The data is arranged according to the binding modes or clusters and provides an overview of selected distances between the quaternary ammonium in cocaine (N^+^) and selected hetero atoms in amino acid residues within the binding site pocket as well as the GlideScore, Emodel, and IFDScore for each pose. The representative pose of **C-I** is marked with a grey shadow. The RMSD given is between the binding mode representative and the current pose. For the outliers the RMSD is relative to the representative of **C-I**.

| **Cluster** | **N^+^-Phe335(O) (Å)** | **N^+^-Asp98(OD) (Å)** | **Asp98(OD)-Tyr176(OH) (Å)** | **RMSD**  **(Å)** | **GlideScore**  **(kcal/mol)** | **Emodel**  **(kcal/mol)** | **IFDScore**  **(kcal/mol)** |
| --- | --- | --- | --- | --- | --- | --- | --- |
| C-I | **2.64** | **3.96** | 3.72 | 0 | -10.41 | -62.54 | -876.09 |
| C-I | **2.79** | **3.68** | 4.29 | 0.97 | -11.89 | -76.45 | -878.43 |
| C-I | **2.83** | **3.39** | 5.13 | 1.40 | -11.83 | -62.67 | -878.28 |
| C-I | **2.69** | **3.79** | 2.84 | 0.50 | -11.89 | -79.91 | -878.15 |
| C-I | **2.60** | **3.36** | 5.11 | 0.56 | -11.31 | -77.50 | -877.68 |
| C-I | **2.73** | **3.84** | 3.86 | 0.72 | -10.74 | -66.66 | -877.58 |
| C-I | **2.72** | **3.76** | 3.69 | 0.57 | -10.92 | -74.79 | -877.54 |
| C-I | **2.63** | **4.43** | 2.88 | 0.60 | -11.42 | -77.42 | -877.32 |
| C-I | **2.91** | **2.91** | 5.21 | 1.22 | -10.83 | -74.32 | -876.44 |
|  |  |  |  |  |  |  |  |
| OUT | 2.90 | 5.27 | 5.80 | 3.03 | -11.98 | -65.05 | -878.63 |
| OUT | 3.01 | 5.46 | 2.84 | 3.49 | -12.36 | -67.32 | -877.90 |
| OUT | 3.04 | 5.27 | 3.67 | 3.16 | -11.27 | -67.12 | -877.25 |
| OUT | 4.10 | 2.74 | 3.72 | 3.93 | -12.14 | -68.59 | -877.08 |
| OUT | 3.99 | 4.54 | 2.80 | 3.63 | -10.06 | -51.87 | -875.01 |
| OUT | 3.07 | 2.76 | 4.15 | 3.68 | -8.17 | -69.35 | -873.55 |
| OUT | 3.40 | 2.70 | 4.47 | 4.05 | -8.08 | -59.49 | -873.48 |
| OUT | 5.82 | 7.08 | 3.90 | 3.89 | -8.00 | -52.79 | -873.31 |
| OUT | 3.31 | 2.87 | 5.05 | 3.90 | -7.50 | -73.91 | -872.94 |
| OUT | 3.47 | 5.09 | 4.88 | 3.08 | -7.65 | -62.86 | -872.09 |
